# Implicit Racial Attitudes Influence Perceived Emotional Intensity on Other-Race Faces

**DOI:** 10.1371/journal.pone.0105946

**Published:** 2014-08-25

**Authors:** Qiandong Wang, Guowei Chen, Zhaoquan Wang, Chao S. Hu, Xiaoqing Hu, Genyue Fu

**Affiliations:** 1 Department of Psychology, Zhejiang Normal University, Jinhua, China; 2 Applied Psychology & Human Development Department, University of Toronto, Toronto, Canada; 3 Department of Psychology, Northwestern University, Evanston, Illinois, United States of America; Ecole Normale Supérieure, France

## Abstract

An ability to accurately perceive and evaluate out-group members' emotions plays a critical role in intergroup interactions. Here we showed that Chinese participants' implicit attitudes toward White people bias their perception and judgment of emotional intensity of White people's facial expressions such as anger, fear and sadness. We found that Chinese participants held pro-Chinese/anti-White implicit biases that were assessed in an evaluative implicit association test (IAT). Moreover, their implicit biases positively predicted the perceived intensity of White people's angry, fearful and sad facial expressions but not for happy expressions. This study demonstrates that implicit racial attitudes can influence perception and judgment of a range of emotional expressions. Implications for intergroup interactions were discussed.

## Introduction

The ability to accurately recognize an individual's emotional state and judge the associated emotional intensity is among the most critical skills for successful social interactions. Specifically, an accurate judgment of others' emotional states can serve as a basis for empathy and inform basic approach/avoidance behavioral tendencies [1]. Due to emotion perception's prevalence and importance during social interactions, an increasing amount of studies have been conducted to understand how people attend to [2], categorize [3], recognize [4], and even mimic facial expressions [5]. On the other hand, given the increasing amount of intergroup interactions which occur in the modern world, an imperative question is whether or not people's attitudes toward other races or out-group members may influence the way they perceive and judge an out-group member's facial expressions. Understanding this question is important because perceiving and judging emotional expressions from an out-group member is a critical part during intergroup interactions and may have significant implications for interventions aimed to foster positive intergroup relations. The present study focused specifically on how people perceive and judge the intensity of a range of positive and negative facial expressions exhibited by out-group members.

Previous research has argued that the ability to perceive basic emotional facial expressions should be universally similar [6], and only biological factors may influence such an ability (e.g., autism is characterized by deficiencies in perceiving emotional expressions [7]). Despite this assumption, recent studies have found that social and cultural factors play an indispensable role in shaping such perception and judgment. For instance, recognizing emotions on other-race faces is more difficult because observers usually have less experience with other-race faces than with their own-race faces [8]. Moreover, previous research consistently finds that one's implicit attitudes toward out-group members influence the extent to which one judges facial expressions [9]–[11]. For instance, in the experiments of Hugenberg and Bodenhausen [9], White participants were presented with video clips depicting Black or White faces changing from an unambiguously angry facial expression (i.e. hostility) to an unambiguously happy one (i.e. friendly) and vice versa. Specifically, White participants who possessed higher implicit anti-Black biases were more likely to report that the angry expression on the Black face stayed longer or appeared quicker than their low-prejudice White counterparts. Subsequent studies provided further evidence revealing that White participants' level of implicit racial bias was associated with the likelihood to categorize angry, racially ambiguous faces (but not happy ones) as Black. Moreover, when racially ambiguous faces were categorized as Black (but not White), White participants with higher implicit biases reported greater intensity of angry facial expression [10], [11]. These findings altogether suggest that people's implicit racial biases exert systematic influences on their perception of out-group members' facial expressions.

However, so far most studies have been conducted in Western culture and focus on White-Black intergroup processes, while little is known about the influence of implicit biases on emotion perception among non-Black out-group cultures. In Western culture, there are pervasive negative evaluations toward Black people [12], [13]. These general negative evaluations are further colored with specific affective responses such as fear and specific beliefs that Black people are hostile, which bias White Americans' perceptions/interpretations of facial expressions displayed on Black people's faces in an aggressive manner [14]. Generalizing this specific White-Black pattern to other ethnic categories, there is some evidence to suggest that people tend to associate out-group members with aggression regardless of the race of out-group members [15], [16]. This suggests a general “out-group = aggressive” evaluative tendency. Based on these prior findings, we investigated whether implicit biases can influence emotional intensity perception among non-Black out-group social categories (e.g., Chinese vs. White). Furthermore, although angry and happy faces may have particular importance in person perception, other emotional expressions such as fear or sadness can play significant roles in guiding people's attention to survival-salient information and empathetic reactions. Therefore, in the present study, we sought to investigate the relationship between implicit racial attitudes and the perception of a range of facial expressions that included not only anger or happiness, but also fear and sadness.

Here, we asked Chinese participants to observe a series of Chinese and White facial expressions (happiness, anger, sadness, fear) and rate each facial expression's emotional intensity. Participants' implicit racial biases were obtained from an evaluative implicit association test (IAT), one of the most widely used implicit tests in studies of intergroup bias [17]. Based on the idea that people have a natural tendency to separate the world into in-groups and out-groups and to favor their in-groups over the out-groups [18], [19], we hypothesized that our Chinese participants would show implicit prejudice toward White people compared with Chinese [15]. Second, based on previous findings that one's implicit racial prejudice can bias the perception of facial expressions from out-group members [9], [11], we also predicted that Chinese participants who possessed a higher level of implicit prejudice would perceive angry (not happy) White faces as more intense than Chinese faces compared with those low in implicit prejudice. Regarding the relationship between implicit prejudice and the perception of other facial expressions, it is possible that implicit prejudice should bias the perception of sad and fearful facial expressions just as they bias the perception of angry facial expression because both sad and fearful expressions are negative in valence like angry expression. However, if implicit prejudice is only associated with threat, then implicit prejudice should only influence the perception of angry and fearful facial expressions because these two emotions are threat-related [20]–[22].

## Method

For our experiment, we have reported all measures, conditions, data exclusions, and how we determined our sample sizes.

### Ethics Statement

**Figure 1:**
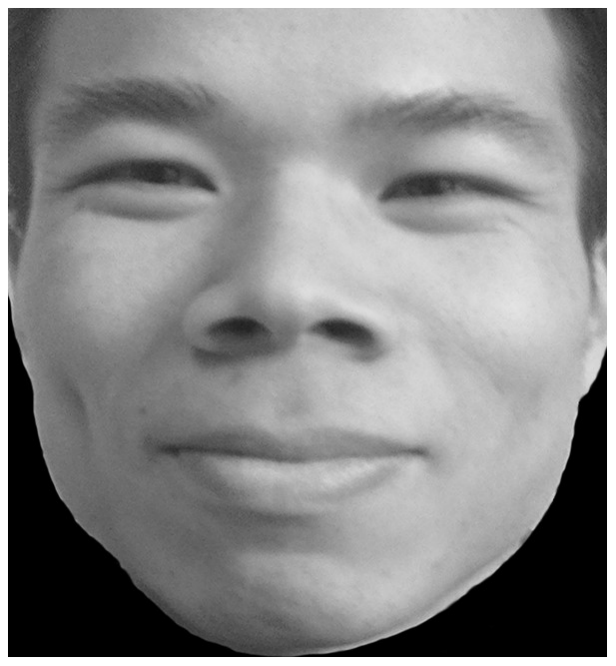
Sample face (Chinese happy face).

**Figure 2:**
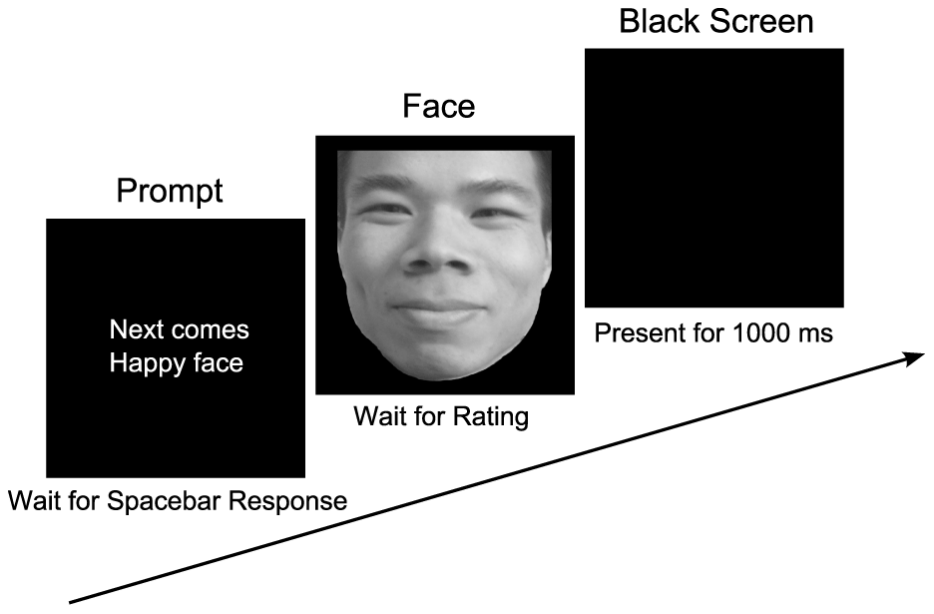
Schema of rating task.

This study was conducted in China according to the NIH research ethics guidelines and received approval from the Zhejiang Normal University Research Ethics Review Committee. Participants gave written informed consent forms prior to their participation and were compensated for their involvement in the study. Participants were ensured that they would come to no harm and were told that they had the option to quit at any time during the experiment with no penalty ensuing. The subjects of the images used in Figures 1 and 2 of this manuscript provided written informed consent, as outlined in the PLOS consent form, to publication of their photograph.

### Participants

Forty undergraduate students (15 males, age 18–23 years old, *M* = 19.53 years, *SD* = 1.31 years) were recruited in the experiment. The sample size was determined based on previous similar studies [9], [10]. All participants were native Chinese and they reported no direct contact with non-Chinese individuals. Data from two additional female participants were excluded: one participant's data from the emotional intensity rating task was lost; The second one's data was discarded because more than 10% of the trials in the implicit association test (IAT) were associated with extremely fast reaction times (<300 ms) [23], which was taken as a sign of non-cooperation.

### Materials

#### Implicit Association Test

To measure implicit attitudes participants hold toward White people, we employed a good-bad evaluative implicit association test (IAT) [23], [24]. This IAT contains two critical double-categorization blocks. In one double-categorization block, participants pressed one button for either positive words (e.g. joy, success) and in-group faces (here, Chinese faces); they pressed another button for either negative words (e.g. cancer, failure) and out-group faces (here, White faces). In another double-categorization block, participants pressed one button for either positive words and out-group faces, and pressed another button for either negative words and in-group faces. If participants held more positive attitudes toward their in-group members than out-group members, such attitudes would be manifested via quicker and more accurate responses during the positive+in-group block than during the negative+in-group block. In the present IAT, target stimuli were five Chinese and five White faces of neutral expression; attribute words were 10 positive words (e.g., “success” and “joy”) and 10 negative words (e.g., “failure” and “pain”). Half of the participants received the Chinese+positive block first whereas the other half of the participants received the White+positive block first.

#### Emotional intensity judgment task

For White and Chinese face photos, there were 16 happy, 16 angry, 16 fearful, and 16 sad expressions (half female in each category). Chinese face photos were selected from Chinese Affective Picture Systems (CAPS) [25] and White faces were selected from KDEF [26] and FACES [27] stimulus set. Each photo's width was set to 600 pixels, and the height was set to 650 pixels. The faces were all normalized to have the same shape and size. All face images were cropped in a frontal view and were rendered grey (see Figure 1). Furthermore, to control the influence of low-level stimulus attributes such as luminance and contrast, all images were matched in overall luminance using Shine Matlab toolbox [28].

### Procedure

Participants arrived at the lab in groups of two or three. After providing informed consent, each participant was seated in front of a computer in an individual cubicle. They were instructed that they would engage in two unrelated tasks that involved word and photo categorizations and rating of the intensity of emotional faces presented in photos.

Participants first completed the IAT [23], [24]. In the second, ostensibly unrelated session, participants finished an emotional intensity rating task. Specifically, this rating task consisted of two blocks, in which either Chinese or White faces were used as stimuli. Half of the participants received the Chinese face block first and the White face block second. The other half of the participants received the blocks in a reverse order. There were 64 photos of either Chinese or White facial expressions in each block. Within each block, each of the photos from four categories of emotions (happiness, sadness, anger, and fear) was randomly presented to the participants. Participants were instructed to rate the intensity of emotions on a series of facial expressions of these four categories.

For this rating task, all stimuli were presented at the center of the computer screen. Each trial started with a prompt indicating which category the following facial expression would be. Participants were instructed to press the space key when they were ready to rate the expressions. A facial expression would then appear on the screen upon the press of the space bar. Participants were instructed to observe the target face carefully and rate the intensity of the expression via a computer keyboard (1 = not intense to 5 = very intense). There was no time limitation for them to rate the expression intensity. Once the participants made a response, a black screen would appear for 1000 ms, after which the next trial began. Schema of experimental design is shown in figure 2.

At the end of the experiment, each participant was fully debriefed and compensated with 10 RMB for their participation.

## Results

### IAT results

We used the D score as an estimate of the IAT effect based on the algorithm developed by Greenwald and colleagues [23]. According to this algorithm, the RT of each error trial was replaced with the corresponding block's mean RT with a 600 ms penalty. A larger D score indicates faster and more accurate performance in the Chinese+Positivity block than in the Chinese+Negativity block, therefore a larger pro-Chinese and anti-White bias. The results suggested that the Chinese participants held an implicit preference for the Chinese over the White people, IAT D = .25 (*SD* = .52), *t* (39) = 3.01, *p* = .005.

### The ratings of the emotional intensity of the Chinese/White faces

A 2 face race (Chinese vs. White)×4 expressions (happiness, anger, fear, and sadness) repeated measures ANOVA was performed on the ratings of the emotional intensity. Note that the adjusted *F* value, *p* value, and degrees of freedom were used henceforth when the Mauchly's Test of Sphericity was significant. The effect of expressions was significant, *F* (2.40, 93.63) = 98.61, *p*<.001, *η2_p_* = .717. The interaction between face race and expressions was also significant, *F* (2.10, 82.03) = 3.96, *p* = .021, *η2_p_* = .092. Pairwise t-tests revealed that both the ratings of emotional intensity for White angry (*M* = 3.10, *SD* = .58) and happy (*M* = 2.76, *SD* = .53) expressions were higher than that for Chinese angry (*M* = 2.95, *SD* = .51) and happy (*M* = 2.59, *SD* = .65) expressions, *t* (39) = 2.16, *p* = .037, Cohen's *d* = .27, and *t* (39) = 2.31, *p* = .026, Cohen's *d* = .29, respectively. Other differences were not significant, all *p*s>.05.

### The relationship between the implicit prejudice and facial expression judgments

Our main analyses focused on the relationship between individual differences in implicit racial attitudes and perceived emotional intensity. Following previous studies focusing on relative rating [29], we subtracted the mean Chinese emotional intensity rating score from the mean White emotional intensity rating score of each expression for each participant. Thus, a higher score indicated higher emotional intensity for out-group faces than for in-group faces. Then, a series of Pearson correlation analyses were conducted between the implicit racial attitudes D scores and the disparity ratings of the emotional intensity of the Chinese and White happy/angry/sad/fearful facial expressions separately.

**Figure 3:**
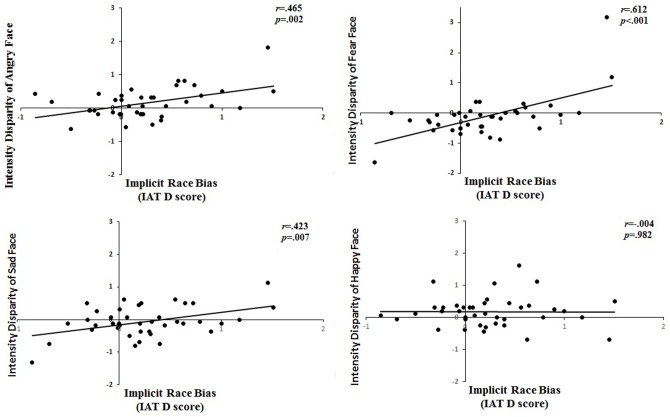
Correlations between the implicit racial attitudes and the disparity rating of the emotional intensity of White and Chinese faces.

Results showed that individual differences in the implicit racial attitudes were significantly correlated with individual differences in rating difference score for an angry expression, *r* (40) = .465, *p* = .002, a fearful expression, *r* (40) = .612, *p*<.001, and a sad expression, *r* (40) = .423, *p* = .007. The correlation for a happy expression was not significant, *r* (40) = −.004, *p* = .982, though. Thus, we found that individual differences in implicit racial attitudes positively predicted the disparity in the perceived emotional intensity of White and Chinese faces for all three negative expressions, but not for the positive happy expression. That is, individuals who had a stronger pro-Chinese and anti-White implicit bias were likely to judge White negative expressions as stronger than Chinese negative expressions. The correlation results are shown in Figure 3.

## Discussion

The present study replicated and extended previous research in examining the relationship between implicit racial biases and perceived emotional intensity of other-race faces in a novel context. We found that even when Chinese participants had no direct contact with White out-group people, they still showed a significant level of implicit pro-Chinese/anti-White prejudice. Most importantly, people who possessed higher level of implicit prejudice tended to judge White people's angry, fearful and sad facial expressions as more intense.

Previous studies that have been conducted in a Western culture setting have consistently showed that a majority of White people hold implicit biases toward out-group members such as Blacks and Muslims [23], [30]. These implicit biases can be acquired and developed via adhering to social norm, or from social learning about the relative status of different social groups [31]–[35]. In the present study, we documented that Chinese participants showed implicit racial biases toward out-group White people compared with their in-group Chinese people. Because the IAT is a relative measure, whether such effect is driven by out-group derogation or in-group favoritism remains to be explored in future studies. However, this pattern of result is clearly consistent with Vaughan and his colleagues' hypothesis that people generally tend to favor the in-groups over the out-groups [19].

Our hypothesis regarding the relationship between implicit racial attitudes and ratings of emotional intensity was supported. Participants' implicit racial biases significantly influenced the perceived intensity for angry, fearful and sad, but not happy facial expressions on out-group White than in-group Chinese faces. Overall, individuals who had a stronger implicit racial prejudice toward White were more likely to judge the negative expressions on White faces as stronger than that on Chinese faces. These results are consistent with previous studies, such as higher implicit prejudice among White Americans was associated with a greater readiness to perceive a threatening expression on Black faces [9]. This relationship holds even when racially ambiguous faces are categorized as Black [11]. Based on these findings, it has been argued that one's implicit racial prejudice is associated with an increased sensitivity to threat-related emotional expressions from African Americans. Moreover, the “other-race = threat” hypothesis [36] would also have predicted that one's implicit prejudice should only influence the perception of threat-related facial expressions. The present study extended previous research in examining the relationship between implicit bias and the perception of a range of negative emotions such as fear or sadness. The results demonstrate that implicit prejudice may be associated with biased perception of general negative emotions that are not only threat-specific. Therefore, our results are more consistent with the assumption that other-race is globally negatively evaluated [37]. Specifically, Hugenberg (2005) found that people were quicker at identifying both angry and sad out-group faces than happy out-group faces [37]. Furthermore, our results suggest that during intergroup emotion perception, it is mainly the valence (positive vs. negative) of emotion, rather than discrete emotions, drives the biased perception [38].

Our results are also consistent with recent cognitive neuropsychology studies. These studies suggest that the amygdala, which is a core neural substrate in emotion processing, is involved in both relatively automatic processing of unpleasant stimuli (especially the perception of anger and fear expressions) [39], [40] and the implicit racial biases as revealed via the IAT [41], [42]. It is likely that the amygdala may similarly mediate the biased perception of negative emotions displayed on other-race faces. Vigilance to threat-related expressions (anger and fear) of out-group/other-race members has significant survival importance for an individual and such vigilance will guide people's initial attention orientation toward out-group members [43]. The present results further suggest that such a threat-related bias may start with vigilance to general negative expressions of out-group/other-race members, which may provide a coarse yet efficient first step that allows the perceivers to have more time to prepare for further adaptive behavior such as fight or flight. This is also consistent with the viewpoint of Adolphs [44] that fear, anger, sadness, and disgust are all subordinate categories to the broader, basic level category of unhappiness. It is plausible that people who are more prejudiced against other-race members are more vigilant toward other-race members' negative emotions in general, which then bias the perceived negative emotional intensities.

We found that implicit racial attitudes had no influences on the rating of emotional intensity for happy facial expression between Chinese and White faces. This result is consistent with previous studies that found that the perception for happiness is not influenced by one's implicit prejudice [11]. Because a large part of intergroup bias is generally grounded in in-group positivity, one would predict that people higher in implicit racial biases may perceive the happiness to be stronger on in-group members' faces than out-group members' faces. However, the present study and other research failed to find this effect. It is not clear why there is no such “in-group  =  happy” effect based on implicit biases. Previous cognitive neuropsychology studies have showed that implicit racial biases as revealed via the IAT are related with activation of the amygdala in response to other-race faces [41], [42]. Thus, it is likely that the positive emotions expressed by in-/out-group faces could reduce amygdala activation in this context, allowing the same ratings to own- and other-race happy faces.

There are some limitations in the present study. For example, because it is difficult to find a stimulus set containing enough Chinese and White emotional faces, the stimuli used here were drawn from different stimulus sets. This selection may introduce confounding factors (e.g. the actors' posed expression intensity). Therefore, such stimulus set difference may lead our participants to rate the emotional intensity to be higher for White angry and happy expressions than for Chinese angry and happy expressions. However, our main goal was to explore the relationship between implicit racial attitudes and the disparity rating of the emotional intensity between White and Chinese faces, not the differences between the two racial faces. Thus, although these faces are selected from different stimulus sets, it is unlikely such a difference should influence our main results.

In summary, we found that Chinese participants' implicit racial prejudice influences the perception of facial expressions on White faces. Specifically, individuals who have a stronger pro-Chinese and anti-White implicit bias were more likely to judge White negative expressions as stronger than Chinese negative expressions. As the world becomes increasingly diversified, perceiving and judging emotional expressions from an out-group member is a critical part during intergroup interactions and may have significant implication for both basic perceptual processes and downstream overt behavior. For instance, perceived higher intensity of anger of an out-group member among high implicit racial prejudice individuals may trigger aggressive behavioral tendencies that can increase intergroup conflict. On the other hand, our results suggest that these individuals also perceived higher intensity of sadness, which may trigger empathetic reactions. If this is true, future studies are necessary to examine when such a perception bias can promote positive intergroup interactions based on empathy, and to develop possible interventions that help individuals to limit the unwanted influence of implicit biases.
